# Regulatory T cells in the skin: Origin, function, and plasticity

**DOI:** 10.1016/j.xjidi.2026.100511

**Published:** 2026-07-17

**Authors:** Yimin Xu, Xinyue Liu, Difeng Fang

**Affiliations:** 1 Laboratory of Molecular Immunology, School of Basic Medical Sciences, Ningbo University, Ningbo, China; 2 Health Science Center, Ningbo University, Ningbo, China

**Keywords:** CCR6, Regulatory T cells, Treg cell migration, Treg cell plasticity

## Abstract

Skin is continually exposed to external stimuli, making this frontline barrier highly susceptible to inflammatory disorders. Foxp3^+^ regulatory T cells (Tregs) are considered guardians of skin homeostasis, with functions that extend beyond immune regulation to include neuroimmunological modulation, hair cycling, and wound healing. In the resting state, skin Tregs originate from the thymus; however, the composition of the Treg population under pathological conditions remains elusive. Notably, emerging studies show that Tregs can acquire proinflammatory characteristics during the development of allergic and autoimmune diseases, raising questions about their fate and function during disease progression. This review discusses the trafficking of Tregs into the skin, their plasticity driven by proinflammatory cytokines and T helper cell lineage-defining transcription factors, and their multifaceted roles in cutaneous immunity. Together, these insights offer a perspective on Treg dynamics in skin health and disease.

## Introduction

The skin, the largest organ of the body, serves as the first line of defense against a wide range of external harmful substances, including physical, chemical, and biological factors. The proper function of the skin depends on the collaboration between diverse immune and nonimmune cells as well as the dynamic balance between the host and microbiota (Gallo, 2017; Nguyen and Soulika, 2019; Zareie et al, 2025). Fine tuning of the immune response and immune tolerance is essential for effective pathogen clearance, wound healing, and tolerance to innocuous substances in the skin. Cutaneous allergic and autoimmune diseases are often regarded as a failure of immune tolerance. Regulatory T cells (Tregs), characterized by the expression of the transcription factor Foxp3 (forkhead box protein P3), are recognized as powerful immune suppressors. They exhibit considerable heterogeneity in origin, transcriptomic profile, TCR repertoire, cytokine production, and effector mechanisms (Dikiy and Rudensky, 2023; Muñoz-Rojas and Mathis, 2021). Both humans and mice lacking functional Tregs develop inflammatory skin disorders (Chatila, 2005; Dudda et al, 2008; Kanangat et al, 1996). Despite the multiple roles of Tregs in maintaining skin homeostasis, their origin, function, and plasticity in the steady state and under inflammatory conditions remain poorly understood. In this paper, we review the current knowledge of skin-resident Tregs in mice and humans, focusing on their tissue-specific molecular characteristics and both immunosuppressive and nonimmunosuppressive functions. In particular, we discuss emerging insights into Treg plasticity and their transdifferentiation potential in physiological and pathological contexts.

## Origin of Skin-Resident Tregs

### Treg development

**Figure 1:**
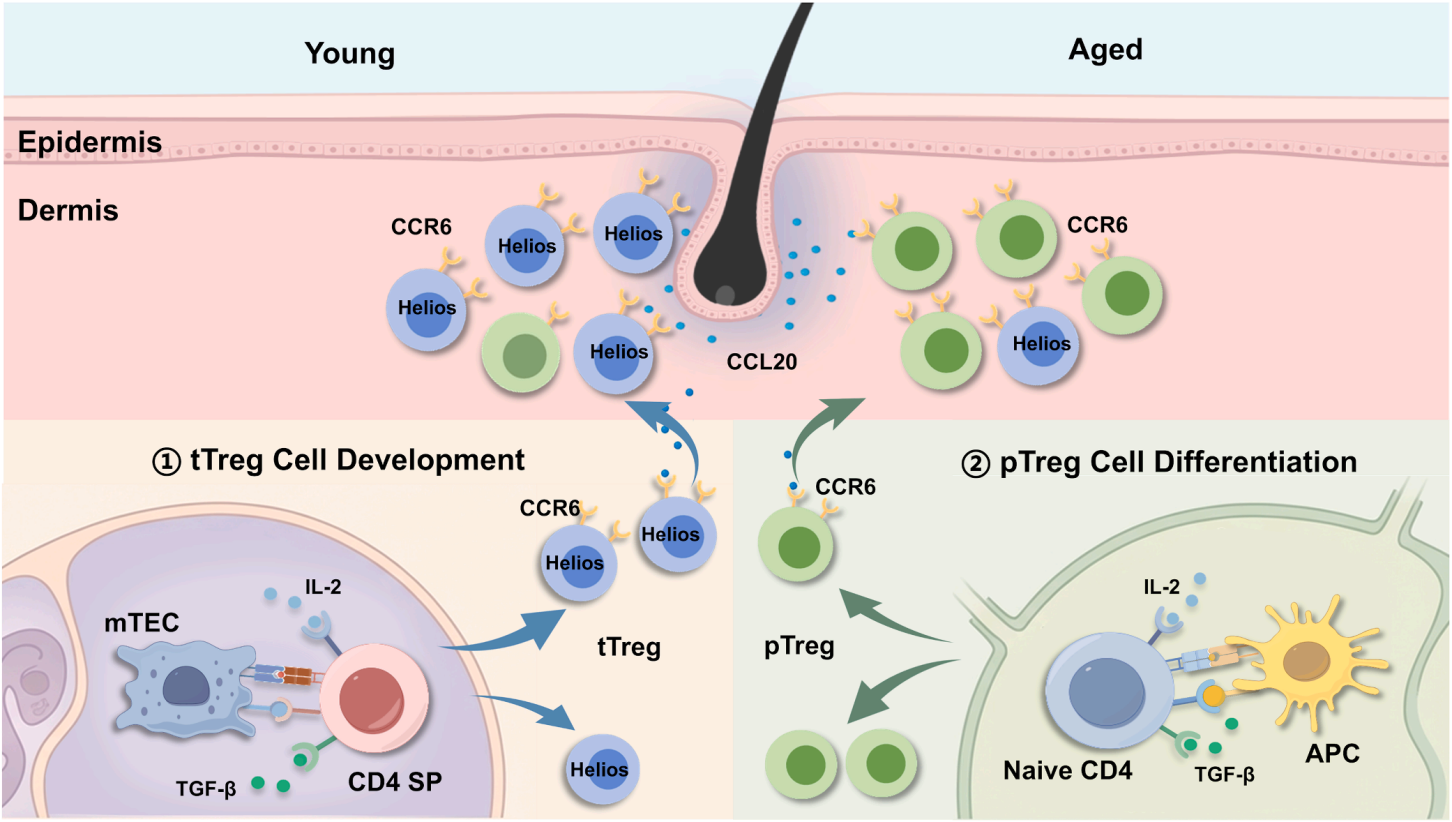
**Origin of skin Tregs.** Skin Tregs originate from 2 main sources: (i) tTregs are first generated in the thymus and migrate to and accumulate in the skin during a defined period of postnatal development; (ii) pTregs differentiate from naive CD4^+^ T cells in peripheral lymphoid organs and migrate to the skin under inflammatory conditions. The expression of chemokine receptor CCR6 is required for the migration of both tTregs and pTregs into the skin in response to CCL20. Given the continuous interaction between the skin and the environment, the ratio of tTregs to pTregs may change with age. APC, antigen-presenting cell; pTreg, peripherally induced regulatory T cell; Treg, regulatory T cell; tTreg, thymus-derived regulatory T cell.

Tregs are usually categorized into 2 major subsets on the basis of their developmental origin in vivo: thymus-derived Tregs (tTregs) and peripherally induced Tregs (pTregs) (Figure 1). tTregs undergo lineage commitment in the thymus, where Foxp3^+^CD25^-^ and Foxp3^-^CD25^+^ CD4 single-positive thymocytes with moderate-to-high affinity for self-peptide–major histocompatibility complexes upregulate Foxp3 and differentiate into the regulatory lineage (Irla, 2022; Jordan et al, 2001; Lio and Hsieh, 2008; Owen et al, 2019). The *Rag2*-GFP transgenic mouse is widely used to track T-cell age, because GFP expression is induced in double-positive thymocytes and repressed upon positive selection. Owing to the half-life of GFP, its expression level reflects the relative age of T cells. Within the mature GFP^-^CD25^+^Foxp3^+^ Treg compartment, a substantial population of CCR6-expressing Tregs has been identified in the thymus of *Rag2*-GFP transgenic mice (Cowan et al, 2016; McCaughtry et al, 2007; Peligero-Cruz et al, 2020). Notably, CCR6 is highly expressed on skin Tregs, suggesting that these cells may originate from the thymus. Whether these CCR6^+^ Tregs represent the long-term retained and more mature tTregs or a population recirculated from the periphery remains to be determined. pTregs are normally differentiated from naïve CD4^+^ T cells into Foxp3^+^ Tregs in peripheral lymphoid tissues in the presence of TGF-β, IL-2, and antigen stimulation. Distinguishing tTregs from pTregs is challenging owing to their significant phenotypic and functional overlap. Whereas tTregs have been identified by the expression of the transcription factor Helios (encoded by *Ikzf2*) and cell surface protein Neuropilin-1 (Dhamne et al, 2013; Thornton et al, 2010; Weiss et al, 2012; Yadav et al, 2012), other studies have shown that pTregs may also express Helios and Neuropilin-1 under certain conditions (Gottschalk et al, 2012; Szurek et al, 2015). This underscores the complexity of distinguishing these 2 populations and the need for reliable markers to do so. Nevertheless, Helios^+^ Tregs make up approximately 70–90% of total skin Tregs (Malhotra et al, 2018), indicating a thymic origin for most skin Tregs at steady state.

### Skin Treg residency

In the skin, Tregs are predominantly localized in the hair follicles, particularly in the infundibulum and isthmus regions. The abundance of Tregs in mouse skin is highly correlated with the hair follicle cycle (Ali et al, 2017). Unique molecular features of skin Tregs, compared with those of their counterparts in other tissues, have been described using single-cell RNA sequencing (Kalekar et al, 2019; Miragaia et al, 2019). In addition, CCR6 is highly expressed in skin and thymic Tregs, in contrast to their counterparts in peripheral lymphoid organs, such as the spleen, small intestine, and lymph nodes (Miragaia et al, 2019; Scharschmidt et al, 2017). In mice, CCR6^+^ tTregs migrate into the skin between postnatal days 6 and 13, a process that is dependent on the CCR6 ligand CCL20 secreted by hair follicle cells (Scharschmidt et al, 2017; Scharschmidt et al, 2015). In T-cell–deficient *Rag*^*−/−*^ mice, adoptively transferred Tregs can migrate into the skin, depending on the expression of CCR6 (Essien et al, 2022). In vitiligo, CCR6-deficient Tregs exhibit impaired migration to the skin, although Treg numbers in the skin-draining lymph nodes are normal (Essien et al, 2022).

Under resting conditions, skin Tregs exhibit high lineage stability, and they do not show noticeable dedifferentiation up to 5 months after tamoxifen-induced transient labeling of Tregs in Foxp3-eGFP-CreERT2×R26-YFP mice (Rubtsov et al, 2010). Interestingly, a later study using the same mouse model but with topical 4-hydroxytamoxifen application to the skin reported that skin Tregs undergo significant attrition over 3 months (Cohen et al, 2024). Nevertheless, it is plausible that tTregs represent the predominant population of skin-resident Tregs early in life, and these cells could be replenished by Tregs from the thymus and/or peripheral lymphoid organs as mice age. This is supported by a human study that shows a higher proportion of Tregs among CD4^+^ T cells in adult skin than in fetal skin (Sanchez Rodriguez et al, 2014). In addition, thymus function declines with age, and tTreg generation may undergo progressive reduction. Therefore, it is reasonable to speculate that the ratio of tTregs to pTregs decreases as mice age, particularly given the lifelong exposure of the skin to diverse environmental challenges. Furthermore, it remains unclear whether these skin-resident Tregs require continuous antigen exposure for survival and whether they can exert innate-like behavior, as observed in certain CD4^+^ memory-phenotype T cells (Kawabe et al, 2017). This is supported by the fact that skin-resident Tregs, having been exposed to strong antigen stimulation, can persist in the skin for more than 30 days (Rosenblum et al, 2011).

Although only a low proportion of Tregs are migratory under resting conditions (Chow et al, 2013), the residency of Tregs becomes much more dynamic during inflammatory states. Tregs are enriched in the dermis and are rare in the hypodermis, but oxazolone treatment significantly promotes Treg accumulation in the hypodermis before the subsequent upregulation of Tregs in the dermis (Chow et al, 2013). In a mouse model of atopic dermatitis (AD) induced by MC903, a synthetic vitamin D3 analog, the frequency of Tregs in both ear skin and peripheral blood declines compared with that in sham controls (Wang et al, 2024). Changes in Treg frequency within the PBMCs of patients with psoriasis remain inconsistent across studies (Furuhashi et al, 2013; Lee et al, 2024). Compared with that in healthy individuals, the proportion of Tregs within the T-cell compartment is reduced in the skin of patients with sarcoidosis and psoriasis (Neuwirth et al, 2025). Nevertheless, an increased frequency and number of Tregs are observed in psoriatic skin in contrast to that in nonlesional skin, and a subset of Tregs produces the T helper (Th)17 cytokine IL-17 (Bovenschen et al, 2006; Keijsers et al, 2013; Sanchez Rodriguez et al, 2014). Therefore, although total Treg numbers may rise in inflamed tissues owing to recruitment from elsewhere, such as the draining lymph node and thymus, the balance between suppressive Tregs and proinflammatory effector cells may become skewed toward the latter.

Unlike those in lymphoid organs, skin-resident Tregs may not require IL-2 for their intrinsic stability. Systemic removal of IL-2 has a subtle effect on skin memory Treg maintenance, whereas IL-7 is required for skin Treg stability (Gratz et al, 2013). Topical treatment of Ub-CreERT2×*Il2*^*flox/flox*^ mice with 4-hydroxytamoxifen to delete *Il2* in the skin reduces Treg numbers in the skin. However, more than half of the Tregs remain, and they express Foxp3 normally (Cohen et al, 2024). One study shows that IL-15 rather than IL-2 stimulates human skin Treg proliferation independently of HLA class II signaling in the presence of fibroblasts (Clark and Kupper, 2007). Thus, skin Treg residency may reflect continuous adaptation to the local microbiota, cytokine milieu, and antigenic environment over time.

### CCL20–CCR6 signaling

CCL20 can be induced in various inflammatory skin conditions. It is rapidly upregulated in keratinocytes during psoriasis (Hedrick et al, 2009; Homey et al, 2000; Kim et al, 2014), but such induction does not occur in vitiligo (Essien et al, 2022). Administration of IL-1α and TNF-α induces CCL20 expression in primary human keratinocytes in vitro, and intradermal injection of these 2 cytokines also evokes CCL20 in mouse skin (Nakayama et al, 2001). Furthermore, CCL20 is strongly increased in the lesional skin of patients with AD compared with that in normal skin (Esaki et al, 2015; Nakayama et al, 2001). Circulating CCR6^+^ Tregs may respond to increased levels of CCL20, migrate to inflamed cutaneous sites, and contribute to the suppression of inflammation.

Currently, the mechanisms underlying CCR6 induction and CCL20-mediated Treg activation are not well-understood. TGF-β is essential for Treg differentiation and maintenance and also strongly induces CCR6 expression on Tregs (Rivino et al, 2010). IL-18 upregulates CCR6 expression in Tregs cultured in vitro (Peligero-Cruz et al, 2020), and IL-18R signaling in Tregs is important for cutaneous immune responses (Alvarez et al, 2023). However, the expression levels of IL-18R on skin Tregs and the identity of the IL-18R^+^ Treg subset remain to be characterized. The costimulatory molecule CD28 also contributes to optimal CCR6 expression in Tregs, and Treg-specific *Cd28*-deficient mice develop spontaneous skin inflammation (Zhang et al, 2013).

CCR6 is a G protein-coupled receptor, and, similar to most G protein-coupled receptors, agonist stimulation leads to receptor desensitization and internalization. In Jurkat cells, CCL20 induces rapid CCR6 internalization, a process that is dependent on C-terminal serine/threonine phosphorylation (Lu et al, 2018). After activation, a portion of internalized CCR6 recycles back to the cell surface, whereas the other fraction is trafficked to lysosomes for degradation. The fate of activated CCR6 appears to be controlled by the phosphorylation status of its intracellular C-terminal domain. CCL20 suppresses Foxp3 expression and promotes the induction of RORγt and IL-17 under Treg polarization conditions in vitro (Kulkarni et al., 2018). It remains unclear whether Tregs residing in the gut and thymus exhibit a similar phenotype upon CCL20 stimulation. Given the notably high surface expression of CCR6 on skin Tregs, the low but constitutive expression of CCL20 in the skin microenvironment may play an important role in maintaining the Treg population. Beyond its well-established chemotactic function, the intracellular signaling pathway downstream of CCL20–CCR6 engagement remains incompletely understood. Further studies are needed to decipher how CCL20–CCR6 signaling modulates Treg function in both physiological and pathological conditions.

In addition, multiple other chemokine receptors contribute to the localization and function of skin-resident Tregs. CCR4 has been shown to regulate the motility of dermal Tregs upon the induction of contact sensitivity (Chow et al, 2013). Skin Tregs utilize the CXCR4–CXCL12 axis to localize to hair follicles and support tissue regeneration (Cohen et al, 2025). Human skin-resident T cells, including Tregs, express CCR4, CLA, CCR8, and CXCR6 as part of their skin-homing profile (Clark et al, 2006). Furthermore, CCR5 expression on Tregs is critical for their proper positioning and suppressive function in the skin to limit autoimmune vitiligo (Gellatly et al, 2021).

## Treg Functions in the Skin

### Immunoregulatory mechanisms of Tregs

The skin is constantly exposed to external insults, and Tregs are essential for maintaining tissue homeostasis through both immune-dependent and immune-independent mechanisms. The functional importance of skin Tregs is evident in inflammatory skin disorders in humans and mice. Individuals with *FOXP3* sequence variant are prone to autoimmune diseases and allergic dysregulation (Bennett et al, 2001; Chatila et al, 2000; Wildin et al, 2001), and alopecia areata has been linked to *FOXP3* promoter polymorphisms (Conteduca et al, 2014). Similarly, Scurfy mice lacking functional Tregs develop severe skin inflammation (Lahl et al, 2007; Sather et al, 2007), and the adoptive transfer of Tregs can partially alleviate inflammation in Scurfy mice (Dudda et al, 2008). The acute depletion of Tregs in neonatal mice also leads to spontaneous skin inflammation (Kim et al, 2007).

Tregs repress the cutaneous type 2 immune response. Skin Tregs express high levels of the transcription factors GATA3 and RORα (Delacher et al, 2017; Miragaia et al, 2019; Sivasami et al, 2023). GATA3 is essential for skin Tregs to repress Th2 cell function during bleomycin-induced skin inflammation (Kalekar et al, 2019). Deleting *Gata3* in Tregs significantly enhances Th2 cell activation, leading to spontaneous skin inflammatory disorders (Harrison et al, 2019; Kalekar et al, 2019). RORα-expressing Tregs have been shown to restrain allergic skin inflammation (Malhotra et al, 2018). Tregs also regulate autoimmune skin pathologies. In both murine and human vitiligo, Tregs restrain CD8^+^ T-cell activity (Essien et al, 2022; Gellatly et al, 2021). A distinct Treg subset depends on peroxisome proliferator–activated receptor γ for skin residency and for controlling imiquimod-induced IL-17A–mediated inflammation (Sivasami et al, 2023). CD25 rather than CTLA-4 is essential for skin Tregs to protect the hair follicle stem cell (HFSC) niche and prevent HFSC-mediated autoimmunity (Cohen et al, 2024). Furthermore, Tregs regulate type 1 immune responses. Under conditions of cutaneous infection with the intracellular parasite *Leishmania*, a T-bet^+^ Treg subset migrates to inflammatory sites and modulates excessive type 1 immune responses, thereby limiting tissue damage (Bourreau et al, 2009).

Mechanistically, Tregs exert immunosuppressive effects through multiple pathways. They secrete anti-inflammatory cytokines, such as IL-10 and TGF-β, which suppress the differentiation and function of myeloid and lymphoid cells. Tregs also promote the maturation of latent TGF-β through integrin αvβ8, which in turn helps to maintain Foxp3 expression and enhances suppressive function (Stockis et al, 2017). In addition, Tregs express ectonucleotidases CD39 and CD73, converting extracellular adenosine triphosphate into adenosine, a potent anti-inflammatory mediator (Jarvis et al, 2021). Furthermore, the high levels of CTLA-4 on Tregs competitively bind to CD80/CD86 on the antigen-presenting cell surface, thereby blocking the transmission of costimulatory signals to effector T cells (Tekguc et al, 2021). Moreover, when Tregs detach from antigen-presenting cells, they can simultaneously remove antigen–major histocompatibility complex complexes from the antigen-presenting cell surface (Zhou et al, 2011). Importantly, by expressing high levels of the IL-2 receptor α chain CD25, Tregs preferentially consume IL-2 in the local environment (Chinen et al, 2016).

**Figure 2:**
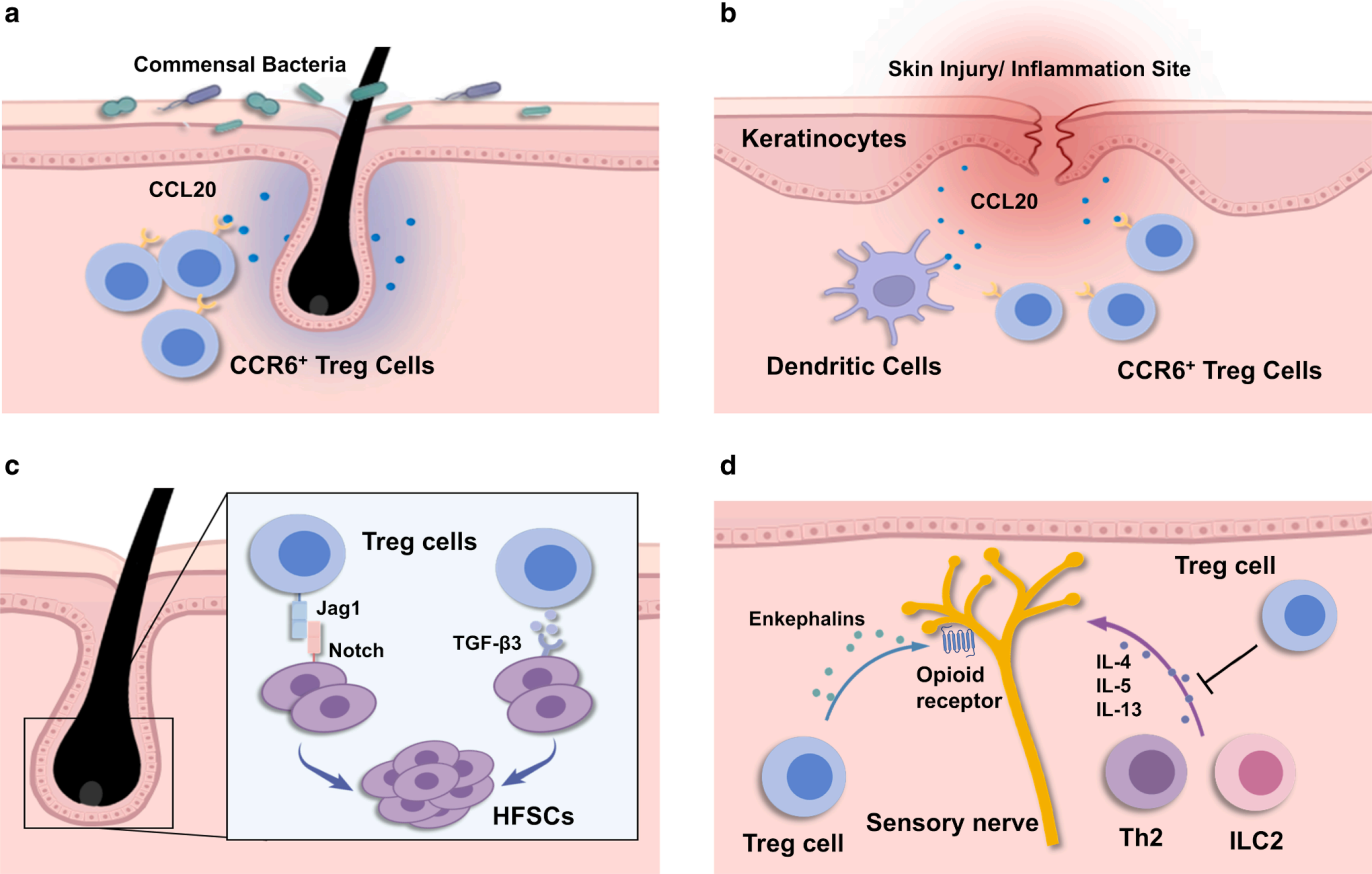
**Functions of skin Tregs.** (**a**) During hair follicle development and bacterial colonization in neonates, CCL20 secreted by the follicular epithelium recruits thymus-derived CCR6^+^ Tregs to the skin, where they contribute to establishing immune tolerance to commensal microbes. (**b**) During skin injury or inflammatory conditions, keratinocytes and dendritic cells secrete CCL20 to recruit circulating CCR6^+^ Tregs to the skin, where they suppress excessive inflammation. (**c**) Tregs facilitate HFSC differentiation and the hair follicle cycle. (**d**) Skin Tregs may regulate nociception through the secretion of enkephalin and the repression of crosstalk between type 2 cytokines and neurons. HFSC, hair follicle stem cell; Treg, regulatory T cell.

Beyond local suppression, skin Treg residency also shapes the skin immune microenvironment. The early wave of Treg migration to the skin is critical for controlling skin inflammation and commensal microbial colonization (Cha et al, 2024; Leech et al, 2019; Scharschmidt et al, 2017; Scharschmidt et al, 2015) (Figure 2a). Tregs that experience antigens in the thymus can persist long term in the skin and maintain tolerance to the same antigen (Rosenblum et al, 2011).

### Tregs in skin regeneration

Tregs play pivotal roles in regulating skin wound healing, hair regeneration, and peripheral neural responses (Figure 2b). Upon skin injury, Tregs not only suppress excessive proinflammatory immune responses—a mechanism crucial for defense against infection—to limit tissue damage but also actively promote tissue repair processes through epithelial regeneration and extracellular matrix remodeling (Boothby et al, 2020; Mathur et al, 2019; Nosbaum et al, 2016). Tregs secrete amphiregulin, which directly stimulates keratinocyte proliferation and accelerates wound healing (Boothby et al, 2020; Nosbaum et al, 2016; Shime et al, 2020; Zaiss et al, 2019). Amphiregulin*,* also activates EGFR signaling in pericytes located in the vascular wall, triggering integrin αv activation and the subsequent release of active TGF-β, thereby promoting pericyte differentiation into myofibroblasts and supporting vascular repair (Minutti et al, 2019). Moreover, autoregulation of amphiregulin secretion and EGFR expression on Tregs helps to establish an immunosuppressive microenvironment conducive to healing during wound repair (Nosbaum et al, 2016). In the later stage of wound healing, Tregs competitively bind to IL-2, thereby reducing IL-4 and IL-13 production by Th2 cells. This effect prevents the overactivation of fibroblasts into myofibroblasts and helps to avoid excessive collagen deposition and pathological scarring (Boothby et al, 2020; Harrison et al, 2019; Kalekar et al, 2019). In addition, Tregs migrate to skin grafts by expressing skin-homing chemokine receptors CCR2 and CCR4, and they inhibit effector T-cell proliferation and suppress the secretion of proinflammatory factors to mitigate graft rejection and prolong allograft survival (Kawai et al, 2021; Steiner et al, 2021).

Tregs regulate the hair follicle cycle by expressing the Notch ligand Jagged1, which activates Notch signaling in HFSCs to promote their proliferation and differentiation, thereby driving the transition of hair follicles from telogen to anagen (Ali et al, 2017) (Figure 2c). Tregs also protect HFSCs from CD8 T-cell attack (Cohen et al, 2024). In addition, glucocorticoid receptor signaling, in concert with Foxp3, promotes TGF-β3 expression in Tregs. This, in turn, activates Smad2/3 signaling in HFSCs to enhance their proliferation and support hair follicle regeneration (Liu et al, 2022). Depletion of Tregs results in increased Th17 cells, which induce keratinocytes to secrete the chemokine CXCL5, leading to excessive recruitment of neutrophils to the injury site. The aberrant neutrophil accumulation impairs HFSC migration and differentiation, ultimately hindering skin barrier regeneration (Mathur et al, 2019).

Recent studies indicate that Tregs are also involved in modulating neuroimmunological responses (Figure 2d). They can regulate nociception through the secretion of enkephalin, a function that is independent of their classical immunosuppressive roles (Mendoza et al, 2025). UVB-activated Tregs promote wound healing through the production of proenkephalin and methionine enkephalin (Shime et al, 2020). Scratching in response to itch damages the skin barrier function and exacerbates allergic skin disease, but scratching benefits the host during *Staphylococcus aureus* infection through a neuroimmune axis (Liu et al, 2025). Type 2 cytokines directly activate itch sensory neurons in both mouse and human AD (Oetjen et al, 2017), and group 2 innate lymphoid cells regulate nerve structure and pain thresholds through the IL-13 signaling pathway (Deshpande et al, 2025). It is interesting to investigate the roles of Tregs in fine tuning the itch–scratch cycle and neuroimmune interaction during the progression of inflammatory skin disorders.

## Treg Plasticity in the Skin

It is recognized that, by expressing the same lineage-defining transcription factor (LDTF) as target Th cells, Tregs can specifically suppress Th cells of the corresponding lineage by adjusting their function and colocalizing in the same niche. Of note, under proinflammatory conditions, Th cell–specific LDTFs can be upregulated in Tregs, driving them to adopt Th cell phenotypes and functions, termed Th-like Tregs (Fang and Zhu, 2017). Consequently, Foxp3^+^ Tregs may lose their suppressive function and acquire a Th cell phenotype during disease development. Some Tregs even transdifferentiate into effector Th cells, termed ex-Treg Th cells (Dominguez-Villar and Hafler, 2018; Fang and Zhu, 2017; Overacre and Vignali, 2016; Zhou et al, 2009). The plasticity and stability of the Treg lineage are closely associated with the pathogenesis of various skin diseases, including psoriasis, AD, autoimmune alopecia, and infection.

### Th2-like Tregs in the skin

**Figure 3:**
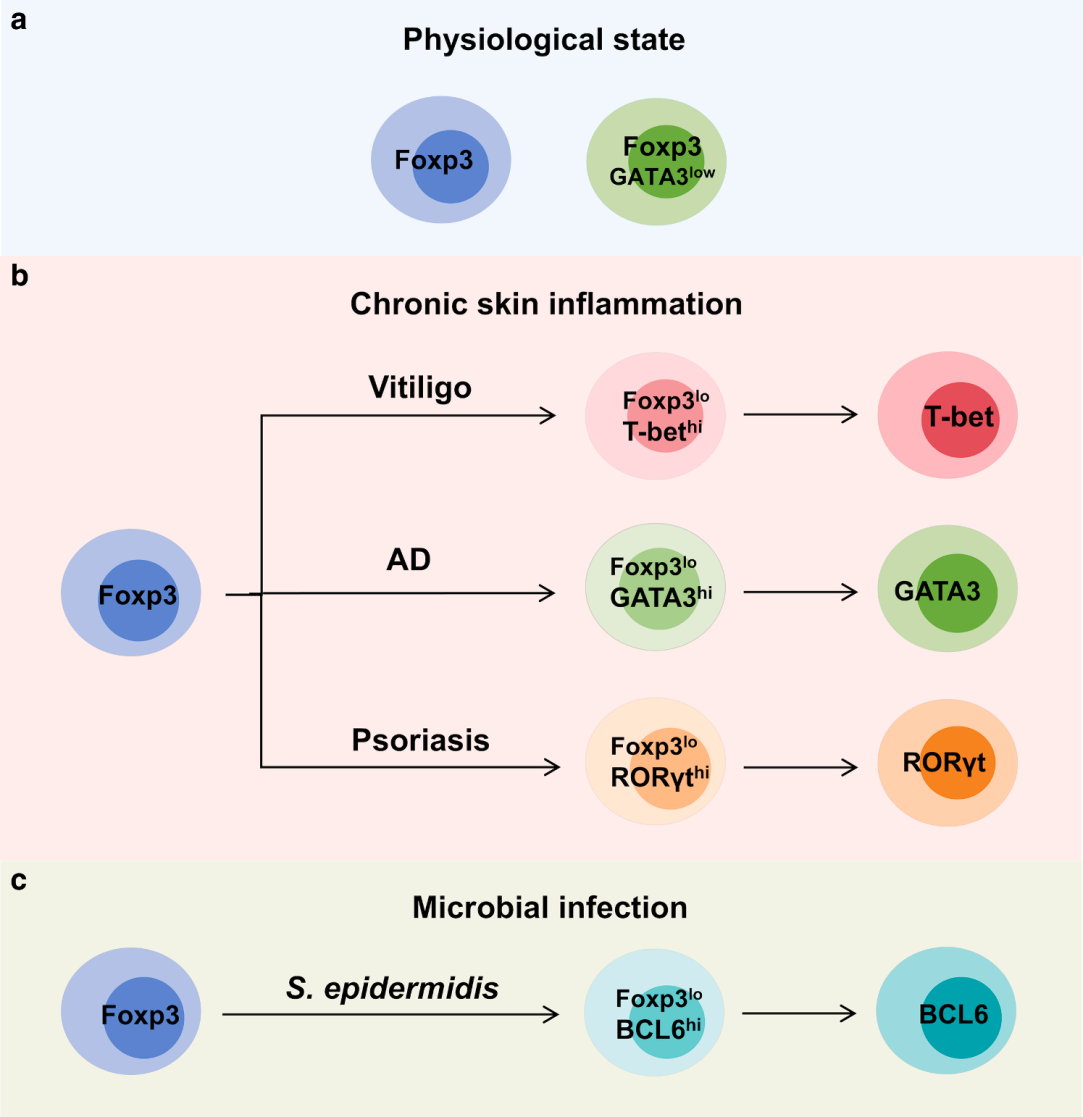
**Plasticity of skin Tregs.** (**a**) Under physiological conditions, Foxp3^+^ Tregs and Foxp3^+^GATA3^+^ Tregs constitute the main populations of skin Tregs. (**b**) In vitiligo, Tregs upregulate the Th1 LDTF T-bet and downregulate Foxp3 expression, acquiring a Th1-like phenotype. Similarly, Tregs express high levels of GATA3 and RORγt in AD and psoriasis, respectively. Those Th-like Tregs may further transdifferentiate into Th cells under inflammatory conditions. (**c**) During *Staphylococcus epidermidis* infection, Tregs may further differentiate into BCL6^+^ Tfh-like cells. AD, atopic dermatitis; LDTF, lineage-defining transcription factor; Tfh, T follicular helper; Th, T helper; Treg, regulatory T cell.

Skin Tregs express group 2 innate lymphoid cell and Th2 cell–related genes, which indicates their potential to respond to stimuli that elicit type 2 immune responses. Under physiological conditions, approximately 80% of mouse skin Tregs and 40% of rhesus macaque Foxp3^hi^CD3^+^ dermal cells express the Th2 cell LDTF GATA3 (Wohlfert et al, 2011), and many GATA3-target genes are expressed by skin Tregs (Miragaia et al, 2019) (Figure 3a). Skin Tregs exhibit high levels of the IL-33 receptor ST2 (Miragaia et al, 2019; Sivasami et al, 2023), and IL-33 stimulation drives these cells to produce the type 2 cytokine IL-13 (MacDonald et al, 2015). MC903 treatment reduces Tregs in mice in a manner dependent on *Il4ra* induction in Tregs (Wang et al, 2024), suggesting that IL-4/IL-13 signaling may either drive the conversion of Tregs into Th2 cells or facilitate their egress from the skin (Figure 3b). In addition, Rbpj-deficient Tregs acquire Th2 cell properties, including open chromatin landscapes and gene expression profiles (Delacher et al, 2019). Notably, Tregs in the skin of patients with the autoimmune disease systemic sclerosis also produce the Th2 cytokines IL-4 and IL-13 (MacDonald et al, 2015).

### Th17-like Tregs in the skin

In human psoriatic skin, a proportion of Tregs acquire a Th17-like phenotype and produce IL-17 (Bovenschen et al, 2011; Kanda et al, 2021; Remedios et al, 2018; Sanchez Rodriguez et al, 2014) (Figure 3b). Within psoriatic lesions, the proinflammatory cytokines IL-6 and IL-23 activate the STAT3–RORγt pathway and repress Foxp3 expression, leading Tregs to acquire a Th17 phenotype. However, this phenotypic shift is not observed in nonlesional skin. Recent work has identified the polyamine-regulating enzyme SSAT1 as a key factor that impairs the ability of Tregs to suppress effector T-cell proliferation and promotes their acquisition of IL-17A–producing capability in the chronic skin inflammatory diseases psoriasis and sarcoidosis (Neuwirth et al, 2025). Overexpression of SSAT1 in Tregs from healthy donors’ skin enhances the expression of IL-17A, IL-2, and RORγt. Pharmacological inhibition of SSAT1 restores Treg number and function, indicating that SSAT1 is a potential therapeutic target for skin inflammatory diseases. In addition, Tregs within the skin lesions of hidradenitis suppurativa also produce IL-17A (Remedios et al, 2018). Furthermore, cutaneous *Candida albicans* infection promotes skin Tregs to produce IL-17A, and the activation of the TNF receptor superfamily members CD27 and OX40 is important for restraining the Th17-like reprogramming of Tregs (Remedios et al, 2018).

### Th1- and T follicular helper–like Tregs in the skin

T-bet^+^ Tregs are present at extremely low frequencies under normal skin homeostasis, yet they can transition into proinflammatory Th1-like Tregs. In vitiligo, these cells are characterized by the acquisition of Th1 cell features, including elevated levels of T-bet, CXCR3, and IFN-γ (Chen et al, 2022) (Figure 3b). Skin-resident Th1-like Tregs exhibit a markedly reduced capability to suppress cytotoxic CD8^+^ T cells recruited to lesions, and they fail to prevent autoreactive T cells from attacking melanocytes, thereby exacerbating immune tolerance breakdown (Chen et al, 2022; Gellatly et al, 2021). BCL6 acts as the core transcription factor governing T follicular helper (Tfh) cell development and identity. Microbial colonization can drive the dedifferentiation of Tregs into Tfh cells, which then support the formation of tertiary lymphoid organs in the skin (Gribonika et al, 2025) (Figure 3c). Similar tertiary lymphoid structures have also been identified adjacent to the tunnels of the chronic inflammatory skin disease hidradenitis suppurativa, and both Tfh cells and Tregs are notably enriched in these structures (Yu et al, 2024). However, whether these Tregs retain suppressive function and whether these Tfh cells originate from Tregs remain unclear.

## Conclusions

Current evidence indicates that tTregs constitute the primary source of skin-resident Tregs, at least during early life. The CCL20–CCR6 axis appears to play a critical role in mediating the migration of tTregs from the thymus into the skin. Supporting this notion, CCR6 expression is extremely high in skin and thymic Tregs, compared with the expression in the Tregs in other tissues. Under inflammatory conditions, the migration patterns and translocation of skin Tregs are markedly altered. Usually, skin-resident Tregs can traffic to draining lymph nodes to repress Th cell differentiation. On the other hand, de novo generated pTregs from lymph nodes or tTregs from the thymus can be recruited into inflamed sites. Notably, activation of CCL20–CCR6 signaling in Tregs leads to CCR6 internalization and degradation, which in turn may result in the exclusion of Tregs from the skin. A deeper understanding of the dynamic balance between Treg recruitment, retention, and egress will be essential for developing targeted immunotherapies for inflammatory skin diseases.

Tregs are endowed with intrinsic immunosuppressive functions, and they are considered a promising immunotherapeutic strategy for restoring immune homeostasis. Tregs exert anti-inflammatory effects in the pathogenesis of AD, psoriasis, and vitiligo. However, Tregs can undergo phenotypic conversion into “pathogenic” or Th-like Tregs that exhibit proinflammatory functions. Therefore, carefully profiling and tracing Treg subsets and phenotypes during disease progression are essential to elucidate their precise roles in skin immune regulation. These observations also raise concerns about the dual-faced nature of skin Tregs. It remains unclear whether certain skin Treg subpopulations serve as “seeds” for proinflammatory Th cells or Tfh cells and whether they contribute to disease initiation. Dissecting skin Treg heterogeneity represents a critical priority in the field of cutaneous immunology.

## ORCIDs

Yimin Xu: http://orcid.org/0009-0003-9546-3277

Xinyue Liu: http://orcid.org/0009-0000-0928-3754

Difeng Fang: http://orcid.org/0000-0003-2105-6743

## Conflict of Interest

The authors state no conflict of interest.
